# Vasoactive Intestinal Peptide Knockout (VIP KO) mouse model of sulfite-sensitive asthma: up-regulation of novel lung carbonyl reductase

**DOI:** 10.1186/1471-2172-12-66

**Published:** 2011-11-21

**Authors:** Anthony M Szema, Sayyed A Hamidi, Antonius Koller, Dwight W Martin

**Affiliations:** 1 Department of Medicine, SUNY Stony Brook School of Medicine, 100 Nicolls Road, Stony Brook, NY, USA, 11794; 2 Department of Pathology, SUNY Stony Brook School of Medicine, 100 Nicolls Road, Stony Brook, NY, USA, 11794; 3 Research Service, Veterans Affairs Medical Center, 79 Middleville Road, Building 61, Room 111C, Northport, NY, USA, 11768

## Abstract

**Background:**

We earlier reported spontaneous features of asthma in Vasoactive Intestinal Peptide knockout mice (VIP KO): 1) peribronchiolar airway inflammation, with accumulation of lymphocytes and eosinophils, 2) pro-inflammatory cytokine production of IL-5, IL-6, with IFN-γ, and 3) airway hyper-responsiveness to inhaled methacholine. In human asthma, a phenotype with sulfite sensitivity leads to airway inflammation and hyper-responsiveness to inhaled sulfites, and is associated with upregulation of anti-oxidant protein lung carbonyl reductase. For the present experiments, we examined the role of VIP in modulating anti-oxidant genes and their proteins, including lung carbonyl reductase.

**Results:**

Four male VIP KO mice and four wild-type age- and gender matched mice had lungs examined for whole genome microarray and a proteomics approach using mass spectrometry. The proteomics analysis revealed that a novel variant of anti-oxidant protein lung carbonyl reductase (car3) was uniquely and markedly elevated in the VIP KO mice. RT-PCR indicated that carbonic anhydrase 3, which is an anti-oxidant protein, was elevated in the VIP KO mice.

**Conclusions:**

These data support the concept that VIP influences the endogenous oxidant/antioxidant balance. One potential implication is that VIP and its analogues may be used to treat inflammatory diseases, including asthma.

## Background

Asthma is an inflammatory airway disease with airway hyper-responsiveness to inhaled irritants such as methacholine and aeroallergens. Vasoactive Intestinal Peptide (VIP) is an endogenous anti-inflammatory peptide found in airway nerve terminals and mast cells, which also has potent airway smooth muscle relaxant properties [1]. We earlier reported that mice lacking VIP have the spontaneous asthma phenotype: 1) peribronchiolar airway inflammation with lymphocytes and eosinophils, and 2) airway hyper-responsiveness to inhaled methacholine. These Vasoactive Intestinal Peptide knockout mice (VIP KO) prepared by disruption by homologous recombination [2,3] have spontaneous airway inflammation, that is chronic, and airway hyper-responsiveness, that does-dependently increases with methacholine in the absence of allergic sensitization and challenge [1]. These features are consistent with the asthma phenotype. There is upregulation of pro-inflammatory and pro-remodeling genes [4]. Th_1 _and Th_2 _pro-inflammatory cytokines (IFN-γ, IL-5, IL-6) are upregulated in bronchoalveolar lavage fluid (BALF) spontaneously--without the need for antigenic sensitization and challenge [1].

In human asthma, a phenotype with sulfite sensitivity, leads to airway inflammation and hyper-responsiveness to inhaled sulfites, and is associated with upregulation of anti-oxidant protein lung carbonyl reductase [5]. Using a combination of genomics and proteomics methodologies, we aimed to determine if genes and their proteins implicated in the pathogenesis of asthma, such as anti-oxidant proteins, are modulated by the VIP gene, suggesting a mechanism of anti-asthma action by VIP. These experiments tested the hypothesis that VIP KO mice compared to wild-type mice have dysregulation of anti-oxidant proteins.

## Methods

### Tissue Preparation

Four male Vasoactive Intestinal Peptide Knockout (VIP KO) mice, ages 4-6 weeks, and four age- and gender -matched wild-type (WT) C57BL/6 mice were anesthetized with pentobarbital (100 mg/kg *i.p*.), and the lungs were removed, placed in polypropylene tubes and quick frozen by immersion in liquid nitrogen. The tissue was stored at -80°C. Subsequently, the tissue was partially thawed to permit dissection of a portion of the lung, the remainder being quick frozen and returned to -80°C. The dissected portion was transferred to a pre-cooled porcelain mortar containing approximately 1 ml of liquid nitrogen (N_2_). The tissue was pulverized with a pre-cooled pestle, and the powdered tissue was transferred to a tared polypropylene tube and weighed. Solubilization buffer --consisting of 7 M Urea, 2M Thiourea, 4% 3-[(3-Cholamidopropyl)dimethylammonio]-1-propanesulfonate (CHAPS), 50 mM Dithiothreitol (DTT) and 1 mM phenylmethanesulphonylfluoride (PMSF)--was added to the powdered tissue at a ratio of 10 μl per mg tissue. The suspension was vortexed with a Vortex Genie^® ^set at maximum speed for 4 × 15 second intervals, with approximately 3 minutes of ice bath cooling of the tube between intervals. The tubes containing the tissue suspension were subsequently sonicated for 10 min. in a Fischer Scientific FS-28 bath sonicator filled with ice-cold water. The tubes were subsequently incubated at 4°C for 2-4 h and centrifuged in a Sorval SM24 rotor at 15,000 rpm, 30 min, 20°C, and the resulting supernatant was transferred to a new polypropylene tube. Aliquots were taken to determine protein concentration, and the residual supernatant was quick frozen in liquid N_2 _and stored at - 80°C. Protein concentration was determined using a modification of the Peterson method [6] that incorporated additional 6% Trichloroacetic acid (TCA) washes to remove DTT, which interferes with the assay. Protein yield was about 0.1 mg/mg powdered tissue.

### 2-DE and Image Analysis

An aliquot of the solubilized protein was further washed using the Bio-Rad ReadyPrep™ 2-D Cleanup Kit according to kit instructions. The resulting pellet was solubilized in 1D buffer containing 7 M urea, 2M thiourea, 4% CHAPS, 50 mM DTT and 0.2% Bio-Rad Biolyte (3-10), centrifuged 5 min, 13,000 rpm to remove any insoluble residuals and the concentration of the solubilized protein was determined as described above. 200 μl of 1D buffer containing 100 μg protein and 0.0002% bromophenol blue was passively equilibrated 17-19 h with a Bio-Rad 11 cm ReadyStrip™ IPG strip, pH range 3-10. The protein was subjected to isoelectric focusing using a Bio-Rad Protean^® ^IEF Cell at a final focusing voltage of 8000 volts and a cumulative 35000 VH. Subsequently, the IPG strips were reduced and equilibrated in SDS for 10 min in Equilibration Buffer I and alkylated for 10 min in Equilibration Buffer II as described in the ReadyStrip™ IPG Strip Instruction Manual (p.19). For the second dimension, the IPG strips were transferred to Bio-Rad Criterion Tris-HCl gel, 8-16% resolving, 4% stacking gel as described in the ReadyStrip™ IPG Strip Instruction Manual. The gels were resolved at room temperature under constant 200 V. Gels were run for each individual mouse (four WT and four KO) as well as pooled samples from WT and KO animals. Pooled samples were formed by mixing equal quantities of protein from each animal in the respective group. The slab gels were stained with 100 ml of SYPRO^® ^Ruby Stain according to the Bio-Rad instruction manual and imaged with a Bio-Rad VersaDoc™ 3000 Imaging System. The resulting images were analyzed using Bio-Rad PDQuest Advanced 2-D Analysis Software, version 8.0. Briefly, the images were analyzed and matched with the following parameters: (1) warp gel images before matching spots; (2) Gaussian model during testing; (3) vertical and horizontal streaking removal; (4) background removal; (5) smoothing with Power Mean filter, kernel size 3 × 3; (6) spot normalization using local regression model

### In-gel Digestion and Protein Identification

Protein spots identified as being unique to WT and KO groups were excised using a Bio-Rad ProteomeWorks™ Plus Spot Cutter with a 1.5 mm cutting tip and delivered to a 96-well plate. Comparable sized excisions of blank regions of the gels were taken to serve as background references during sample processing. The excised gel pieces were destained, reduced, aklyated and digested with trypsin (Promega Gold, Mass Spectrometry Grade), essentially as described by Shevchenko et al. (1996) [7] with minor modifications. The resulting concentrated peptide extract was diluted into a solution of 2% Acetonitrile (ACN), 0.1% Formic Acid (FA) (Buffer A) for analysis.

The peptide mixture was analyzed by automated microcaplillary liquid chromatography-tandem mass spectrometry. Fused-silica capillaries (100 *μ*m i.d.) were pulled using a P-2000 CO_2 _laser puller (Sutter Instruments, Novato, CA) to a 5 μm i.d. tip and packed with 10 cm of 5 *μ*m Magic C18 material (Agilent, Santa Clara, CA) using a pressure bomb. This column was then placed in-line with a Dionex Ultimate 3000 equipped with an autosampler. The column was equilibrated in buffer A, and the peptide mixture was loaded onto the column using the autosampler. The HPLC pump flowed at 100 *μ*L/min, and the flow rate to the electrospray tip was reduced to ~ 200-300 nL/min by a split. The HPLC separation was provided by a gradient between Buffer A (2% ACN, 0.1% FA) and Buffer B (98% ACN, 0.1% FA). The HPLC gradient was held constant at 100% buffer A for 20 min after peptide loading followed by a 30-min gradient from 0% buffer C (100% Buffer A) to 40% buffer B. Then, the gradient was switched from 40% to 80% buffer B over 3 min and held constant for 3 min. Finally, the gradient was changed from 80% buffer B to 100% Buffer A over 1 min, and then held constant at 100% Buffer A for 40 more minutes. The application of a 1.8 kV distal voltage electrosprayed the eluted peptides directly into a Thermo Fisher Scientific LTQ XL ion trap mass spectrometer equipped with a nanoLC electrospray ionization source (ThermoFinningan, San Jose, CA). Full mass spectra (MS) were recorded on the peptides over a 400-2000 *m*/*z *range, followed by five tandem mass (MS/MS) events sequentially generated in a data-dependent manner on the first, second, third, fourth and fifth most intense ions selected from the full MS spectrum (at 35% collision energy). Mass spectrometer scan functions and HPLC solvent gradients were controlled by the Xcalibur data system (ThermoFinnigan, San Jose, CA).

MS/MS spectra were extracted from the RAW file with Readw.exe (http://sourceforge.net/projects/sashimi).

The resulting mzXML file contains all the data for all MS/MS spectra and can be read by the subsequent analysis software. The MS/MS data was searched with Inspect ^2 ^against a mouse IPI database (Ver. 3.43) with optional modifications: +16 on Methionine, +57 on Cysteine, +80 on Threonine, Serine and Tyrosine. Only peptides with at least a p value of 0.01 were analyzed further. Common contaminants (e.g. keratins) were removed from the data. Proteins identified by at least 3 distinct peptides within a sample were considered valid; when sample signal was very weak, 2 distinct peptides were accepted for a valid identification.

### Whole Genome Agilent Microarray Analysis

Five VIP KO and 5 wild-type mice were anesthetized and lungs were removed, flash frozen in liquid nitrogen and sent to SAB Biosciences for analysis. Microarray data were collected using the Whole Mouse genome (4 × 44 K) oligo microarray kit with Sureprint technology (Agilent Technologies 395 Page Mill Rd, Palo Alto, CA 94306). These arrays contain probes for 41,000+ mouse genes and transcripts represented, all with public domain annotations. The enriched array content sourced from UCSC, RefSeq, RIKEN, NIA, Ensembl, UCSC Goldenpath, and Unigene databases. Over 70% of the represented probes are validated by Agilent's laboratory validation process.

### RNA extraction

The lungs were homogenized in 2 ml of TRIzol, using a Polytron homogenizer (VWR). RNA extraction was done following the TRIzol protocol (Invitrogen, Carlsbad, CA, Cat. # 15596-026).

### RNA quality control

RNA samples were run on the Agilent Bioanalyzer. The integrity of RNA was assessed by looking at 18 & 28s rRNA peaks and by the RNA integrity number (RIN). We also took the concentration of RNA on the nano drop and made sure that the 260/280 ratio was above 2.0 and the 260/230 ratio was above 1.7.

Total RNA (0.6 μg) from 6 samples were reversed transcribed into cDNA using T_7_dT_24 _primer containing the poly T sequence and the promoter for T7 RNA polymerase. After second strand synthesis, double-stranded cDNA was used as the template to generate complementary RNA (cRNA) in a modified "Eberwine" type RNA amplification process. During this amplification process, cRNA was labeled with aminoallyl-UTP (Sigma). 8 μg Aminoallyl-cRNA each from 8 mouse samples was conjugated with Cy3-NHS ester (GE Healthcare) to generate Cy3-labelled cRNA.

Before each microarray hybridization, 1.65 micrograms of Cy3-labelled cRNA was added to cRNA fragmentation mix. After the fragmentation reaction was stopped by adding hybridization buffer, the mixture was loaded onto a Whole Mouse Genome Oligo Microarray assembly.

Hybridization was done at 65°C and 4 rpm for 17 hours. After that, the Whole Mouse Genome Oligo Microarray assembly was dissembled in hybridization wash buffer 1 (2× SSC, 1% SDS) at room temperature. The microarray slides were washed in fresh hybridization wash buffer 1 for 1 minute, followed by 1 minute in 37°C hybridization wash buffer 2 (0.1× SSC, 0.5% SDS), 1 minute in room temperature acetonitrile and 30 seconds in the stabilization and drying solution.

The dried microarray slide was scanned into a TIFF image file on an Agilent microarray scanner at 5 μm resolution using default settings. Microarray intensity data were extracted from the TIFF image using Agilent Feature Extraction Software 9.1.3.

### Data Analysis

The raw data file containing raw intensities was exported to Gene spring Software GX. We eliminated data for all the control probes and data from genes which were absent in all the samples. We further narrowed down the list by retaining genes whose expression differences among 2 groups are more than 2 fold and a p value cut off of less than 0.05.

## Results

Lung tissue was dissected from wild type C57BL/6 (WT) and VIP knock-out (KO) mice and the resultant cell lysate was subjected to two-dimensional gel electrophoresis as described in the METHODS section. Approximately 650 spots were detected per gel. Statistical analysis using the Student's t-test algorithm in the PDQuest software indicated 64 spot variations between KO and WT groups. Qualitative analysis indicated 3 spots (minimum 10 fold over background) in WT that were not in KO and 3 spots in KO that were not in WT. We focused our protein identification analysis on these spots.

**Figure 1:**
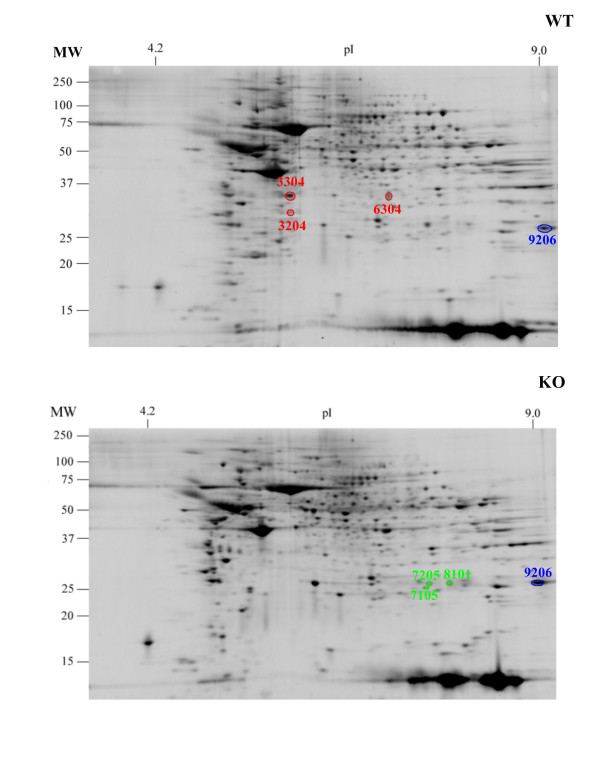
**Two-dimensional gel electrophoresis of lung tissue samples obtained from wild type (WT) and VIP KO (KO) mice**. The above are typical images obtained from gels of both tissue types. The staining intensities of the protein spots were analyzed using BioRad PDQuest software. The three spots (3204, 3304 and 6304) in the WT gel were significantly and substantially under-expressed in the KO gel. The three spots (7105, 7205 and 8101) in the KO gel were significantly and substantially over-express in the KO gel. Spot 9206, encircled in both gels was expresses at essentially equal levels in both gels.

Figure 1 shows Sypro Ruby stained two dimensional polyacrylamide gels of proteome extracts from wild type (WT) and knockout (KO). Analysis of spot optical density using PDQuest software, as described in METHODS, indicated a small number of protein spots with density differences between the two samples. Sample spot protein (SSP) numbered 3204, 3304 and 6304 are circled in Figure 1A and were either absent or had a much lower optical density in the KO sample. SSPs numbered 7105, 7205 and 8101 are circled in Figure 1B and were determined to be absent in the WT sample.

**Figure 2:**
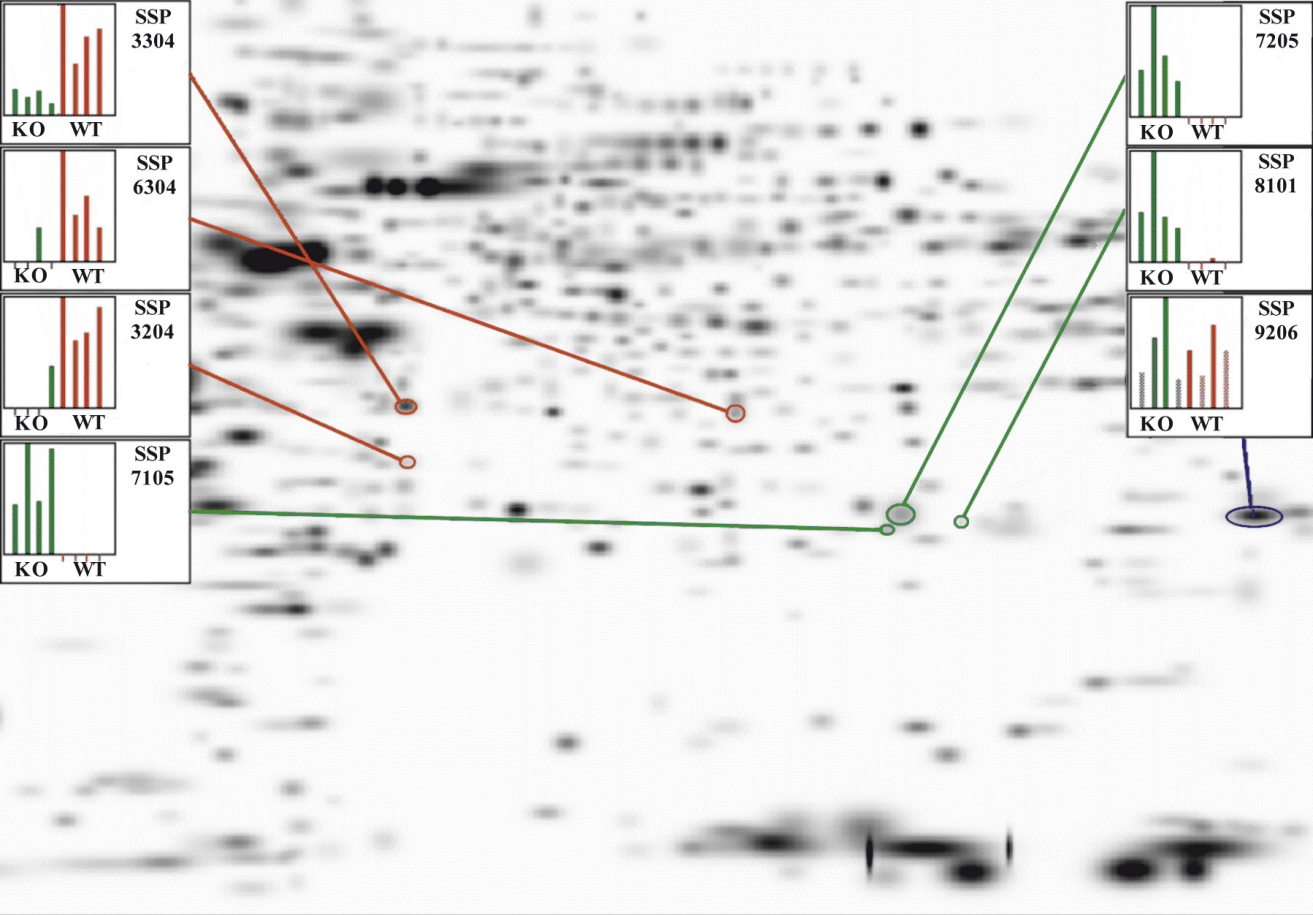
**Master gel generated in PDQuest analysis**. Inserts have histographs of the relative intensity of each spot in each of the eight gels under study. The hatched bars in the histographs for SSP 9206 indicate optical saturation.

Figure 2 shows the master gel image, a composite gel constructed by the PDQuest software, that contains all significant spots from the eight gels used in this study. The spots of interest are indicated and the inserts display histographs of spot optical density. Each histograph bar represents the optical density of the respective spot in each of the eight gels. Table S1 in the Additional file 1 gives the numeric values for the spot densities in each of the gels. The same spots were excised from each gel, pooled, subjected to in-gel digestion with trypsin and the resulting peptides analyzed by LC/MS/MS as described in METHODS. When more than one protein was identified in a spot digest, the predominant protein from each spot was determined based upon the spectral count, the number of peptides identified and the coverage of the protein. To further eliminate ambiguity in spot identification, the composition of comparable regions in both KO and WT gels were compared. The results of these determinations and a summary of spot optical density quantification are given in Table 1.

**Table 1:** Summary of SSP optical density and LC/MS/MS determined protein identity of the excised spots

SSP#	Protein	Symbol	IPI Accession #	spectra	peptides	MW(kDa)/pI calculated	MW(kDa)/pI observed^a^	Av^c ^KO OD	Av WT OD	KO/WT	WT/KO	p value (t-test)
**7105**	Triosephosphate Isomerase	Tpi1	00467833	8	4	26.71/6.9	25.6/7.4	39301	0	∞	0	0.017
**7205**	Dihydropteridine Reductase	Qdpr	00459279	5	3	25.57/7.7	27.1/7.5	222665	0	∞	0	0.032
**8101**	Carbonyl Reductase	Cbr2	00128642	15	7	25.96/9.1	25.8/7.8	210771	2855	73.8	0.01	0.044
**3304**	Pyruvate Dehydrogenase	Pdhb	00132042	36	13	38.94/6.4	37.1/5.6	72099	312442	0.23	4.33	0.011
**3204**	Superoxide Dismutase	Sod3	00114319	12	4	27.39/6.4	32.8/5.6	10569	90285	0.12	8.54	0.002
**6304**	Glycerol-3-Phosphate Dehydrogenase	Gpd1	00230185	16	8	37.57/6.8	36.6/6.9	19546	148182	0.13	7.58	0.038
**9206**	Carbonyl Reductase	Cbr2	00128642	33	16	25.96/9.1	25.96/9.1^b^	546335	521831	1.05	0.96	0.905

^a ^Based on internal benchmarks.

^b ^Served as one of four internal benchmarks.

^c ^Average optical density of spots from four gels.

KO/WT and WT/KO are ratios of average optical densities and the t-Test p-values are two-tailed, unpaired determinations from four gels of each type.

As indicated in Figure 2 and quantified in Table 1, SSP numbers 3204, 3306 and 6304 have higher optical density in WT samples with WT/KO optical density ratios of 8.5, 4.3, and 7.6, respectively, suggesting that the expression of the proteins responsible for these spots may be down regulated or suppressed in the KO samples. SSP 3304 was composed of, in the main, pyruvate dehydrogenase, guanidine nucleotide-binding protein and annexin-3. SSP 3204 was mainly composed of superoxide dismutase, annexin 3 and chloride intracellular channel protein 5. To further resolve the complexity of these spots we examined the protein composition in the regions of the KO gels that correspond to these spots. Analysis of the 3304 spot location in KO gels revealed the presence of comparable amounts of guanidine nucleotide-binding protein and annexin-3, but no pyruvate dehydrogenase. Likewise, analysis of the 3204 spot location in KO gels indicated that superoxide dismutase was only present in this location in the WT gels. We therefore conclude that the increased optical density in SSP 3304 and 3204 are due the presences of pyruvate dehydrogenase and superoxide dismutase, respectively. SSP 6304 was identified as glycerol-3 phosphate dehydrogenase. We also see in Figure 2 that SSPs 7105, 7205 and 8101 have higher optical density in the KO samples and are essentially absent in the WT gels (quantified in Table 1) indicating that the proteins responsible for these spots are up-regulated in the KO tissue. SSP 7105 was identified as triosphosphate isomerase, SSP 7205 was identified as dihydropteridine reductase and SSP 8101 was identified as carbonyl reductase.

**Figure 3:**
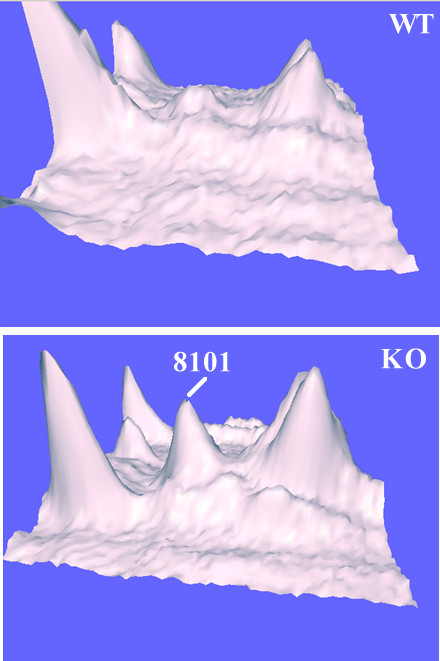
**Three-dimensional optical intensity profile of the region surrounding SSP 8101 in the KO gel and the corresponding region in the WT gel**. The profile was generated by the PDQuest software.

As shown in Table 1, the theoretical molecular weight (MW) and pI for most of the above proteins agree reasonably well with the observed values. The one major exception was SSP 8101. A 3-dimensional pixel density profile of the region encompassing SSP 8101 in the WT and KO gels of Figure 1 is shown in Figure 3, indicating the location of SSP 8101. Carbonyl reductase has a theoretical MW and pI of 25,960 and 9.1 respectively. SSP 9206, its location indicated in Figures 1 and 2, was identified as carbonyl reductase by LC/MS/MS analysis and found to be of equal abundance in both KO and WT samples (Table 1). Spot 8101 therefore results from an isomer of carbonyl reductase that is significantly abundant in KO tissue but essentially absent in WT. As noted in Table 1, Spots 8101 and 9206 migrate in the gel with essentially identical M_r _indicating a change in pI without a substantial change in protein mass. There are four isomers of carbonyl reductase (Cbr1-4). The greatest sequence identity exists between Cbr1 and Cbr3. Cbr2 has <30% sequence identity or similarity with the other isomers. All carbonyl reductase peptides identified in spot 8101 were unique to Cbr2.

The following proteins were upregulated in VIP KO mice: lung carbonyl reductase, Triosephosphate isomerase, Dihydropteridine reductase.

**Lung carbonyl reductase (SSP 8101 and 9206)**(Cbr2) is an anti-oxidant protein responsible for lung protection against inflammation and fibrosis^3^. Another strain of mice (A/J) is resistant to lung inflammation and fibrosis at low levels of ionizing radiation. These A/J mice strongly upregulate five anti-oxidant proteins (superoxide dismutase 1 (Sod1), cytochrome c oxidase, glutamate dehydrogenase, biliverdin reductase, peroxiredoxin and carbonyl reductase) in lung, in response to exposure to ionizing radiation^3^. In contrast, wild-type C57BL/6 mice up-regulate only one of these 5 anti-oxidant proteins (lung carbonyl reductase 2) and have lung inflammation and fibrosis at low doses of radiation exposure.^3 ^The VIP KO mice are backcrossed to C57BL/6. While there are minimal changes in genomic expression of these 5 proteins, there is strong expression of a lung carbonyl reductase isomer that has an altered isolectric point shifted from pI 9.1 to pI 7.8. It is unclear at this time whether the altered pI is due to a translational or post translation modification. Given that the gene for lung carbonyl reductase was not significantly changed in expression between VIP KO and WT mice (Table 2), this would tend to support a mechanism of posttranslational modification in VIP KO mice.

**Table 2:** Summary of gene expression levels obtained from whole genome microarray analysis of WT and KO lung tissue samples.

Gene	Symbol	Average KO	Average WT	KO/WT Fold change	P value
triosephosphate isomerase 1	Tpi1	1.15	0.89	1.29	0.033
quininoid dihydropteridine reductase	Qdpr	0.93	1.09	0.85	0.195
pyruvate dehydrogenase	Pdhb	1.05	0.95	1.10	0.065
superoxide dismutase 1	Sod1	0.82	1.11	0.74	0.017
superoxide dismutase 2	Sod2	0.92	1.04	0.88	0.041
superoxide dismutase 3	Sod3	1.09	0.98	1.11	0.543
carbonyl reductase 2	Cbr2	1.00	1.02	0.97	0.630
carbonyl reductase 3	Cbr3	1.00	1.19	0.84	0.439
carbonic anhydrase 3	Car3	1.67	0.39	4.35	0.001
glycerol-3-phosphate dehydrogenase 1	Gpd1	1.83	0.66	2.76	0.095
glycerol-3-phosphate dehydrogenase 2	Gpd2	1.15	0.88	1.30	0.009

All of the above gene expression levels were confirmed by Real-Time PCR analysis (data not shown).

**Triosephosphate isomerase (SSP 7105, Tpi1)**was up-regulated in all 4 VIP KO mice. There were no detectable SSP 7105 spots in the gels from all four WT mice (p value = 0.017, Table 1). Tpi gene was 1.29 fold increased in VIP KO vs. WT with a p value of 0.03. (Table 2).

**Dihydropteridine reductase (SSP 7205, Qdpr)**was strongly up-regulated in all four VIP KO mice. There was no detectable SSP 7205 spots in any of the four WT mice (p value = 0.0318, Table 1).

The following proteins were downregulated in VIP KO mice: Pyruvate dehydrogenase, Extracellular superoxide dismutase, Glycerol-3-phosphate

**Pyruvate dehydrogenase (SSP 3304, Pdhb)**E1 component subunit beta, mitochondrial precursor catalyzes conversion of pyruvate to acetyl coA and Co2 (Krebs cycle). This enzyme was down-regulated in the VIP KO mice with a WT/KO optical density ratio of 4.33 (Table 1).

**Extracellular superoxide dismutase (SSP 3204 Sod3)**is an anti-oxidant protein that destroys radicals which are normally produced within cells and which are toxic to biological systems; it has NMDA receptor associated protein activity. The optical density of SSP 3204 (Sod3) is substantially reduced in the gels from the KO VIP mice (WT/KO = 8.54, Table 1). The genome microarray data indicated no significant change in Sod3. However, mRNA levels of both Sod1 and Sod2 were somewhat reduced in the KO VIP mice with p values of 0.17 and 0.41, respectively (Table 2).

**Glycerol-3-phosphate (SSP 6304, Gpd1, cytoplasmic)**is decreased in VIP KO mice with a WT/KO optical density ratio of 7.58 (Table 1). Gpd participates in the pentose monophosphate shunt and leads to production of oxygen free radicals. Perhaps down-regulation is a protective mechanism. Our genome array studies (Table 2) indicated no significant change in Gpd1 and that Gpd2 (mitochondrial) is up-regulated slightly (KO/WT = 1.3, p value = 0.009). There is no sequence overlap between Gpd1 and Gpd2 and it is unclear whether the up-regulation of Gpd 2 is compensatory to the down-regulation of Gpd1.

In the genome array studies, the gene for Carbonic anhydrase was highly upregulated in the KO mice. However, the protein was not identified in the proteomics experiment, so we don't know if the protein is not modulated or if the protein is actually present.

**Carbonic anhydrase (Car3)**is increased significantly (p value = 0.001, Table 2) with a 4.35 fold change on gene microarray. Car3 catalyzes reversible hydration of carbon dioxide leading to production of H_2_O_2 _and this may account for increased peroxidation in VIP KO mice.

## Discussion

The following three protein were upregulated in VIP KO mice and may modulate the asthma phenotype:.

### Lung carbonyl reductase

Although we did not test the function of lung carbonyl reductase in VIP KO mice, this different form may have a bearing on function since the asthma phenotype is present despite strong expression of the isomer. Lung carbonyl reductase is essential in pulmonary metabolism of endogenous compounds such as aliphatic aldehydes and ketones derived from lipid peroxidation (anti-oxidant), 3-ketosteroids and fatty aldehydes and is induced by glucocorticoids and activated by fatty acids [8]. The presence of an abnormal isomer may have a significant impact on lung function.

This may be significant in asthma. Recent studies indicate that there is an increase in reactive oxidant metabolites in acute exacerbations of asthma [9]. These authors studied 42 outpatients with acute exacerbations of asthma, *vs*. 11 outpatients with a stable asthma *vs*. 40 healthy humans. Serum reactive oxygen metabolite levels were significantly higher in acute asthma exacerbations *vs*. stable asthma *vs*. healthy controls. Serum eosinophil cationic protein and plasma polymorphonuclear elastase were increased in acute exacerbations and moderately correlated to reactive oxygen metabolite levels. Reactive oxygen metabolite levels decreased with systemic steroids and bronchodilators.

Since our mice have the spontaneous asthma phenotype, and asthma and its exacerbations are associated with oxidation and reactive oxygen metabolites, then an alteration in the isomeric make-up of anti-oxidant proteins could explain why this asthma in VIP KO mice does not spontaneously resolve. The normal endogenous counter-regulatory mechanism against asthma may not be intact or may be disrupted by the presence of an abnormal isomer. In fact, the asthma pathology worsens with age in VIP KO mice, leading to increased inflammation over time which is also consistent with an accumulation of abnormal isomer with age.

With oxidants including reactive oxygen species being implicated in the pathogenesis of asthma, VIP's mechanism of action in reversing the asthma phenotype would be its anti-oxidant effect. These data support the concept of using VIP as an asthma drug. Other authors have reiterated the ample evidence of oxidative stress in asthma but have highlighted the limited information regarding endogenous anti-oxidant defense systems. Sackesen and colleagues noted decreased glutathione peroxidases and superoxide dismutase --significantly lower--in asthmatic children *vs*. controls [10]. In our VIP KO mice, superoxide dismutase was strongly downregulated in protein expression. Since childhood asthma is associated with significant decreases in various components of both enzymatic and nonenzymatic anti-oxidant defenses, it follows that VIP KO mice with the spontaneous asthma phenotype also lack or have reduced anti-oxidant proteins (superoxide dismutase) or have abnormal forms of anti-oxidant proteins (lung carbonyl reductase).

VIP therefore appears to contribute to modulation of the oxidant/antioxidant balance in asthma, since wild-type mice with VIP have functional lung carbonyl reductase and no spontaneous asthma, whereas VIP KO have abnormal carbonyl reductase and have spontaneous asthma. A glutathione gene variant GSTP1 has been linked as a susceptibility gene for childhood asthma [11]. It follows that pro-oxidant states are pro-asthmatic and endogenous mechanisms that are anti-oxidant, such as VIP, which scavenges singlet oxygen, are potently anti-asthmatic.

### Triose phosphate isomerase

Tpi is important in carbohydrate biosynthesis and gluconeogeneis and glycolysis. This may lend support to the concept of diabetes in VIP KO mice. Of relevance to the asthma model is that triose phosphate isomerase and glyceraldehyde 3- phosphate 3 dehydrogenase were the second and third most upregulated proteins in a proteomics analysis of lungs from inflamed ovalbumin sensitized and challenged C57BL/6 mouse model of asthma [12]. If there is a limited supply of oxygen, cells are dependent on the glycolytic pathway for anaerobic ATP production; many of the glycolytic enzymes are upregulated during hypoxia.

### Dihydropterine reductase

Dihydropteridine reductase is a product of enzyme tetrahydrobiopterin (BH-4) which is an essential cofactor for phenylalanine, tyrosine, tryptophan hydrolase. It is important for eNOS production. Tetrahydrobiopterin is a key factor in the production of nitric oxide in mast cells [13]. Nitric oxide synthase (NOS) has obligate requirements for BH4. Increased levels of dihydropteridine reductase would be consistent with increased endogenously produced NO by mast cells, which is consistent with the spontaneous high IgE levels in VIP KO mice and the asthma phenotype (Szema, unpublished data).

The following three protein were downregulated in VIP KO mice:

### Pyruvate dehydrogenase

Decreased glycolysis would mean decreased available ATP. Perhaps this may explain impaired thermogenesis in VIP KO mice.

Decreased pyruvate dehydrogenase may be important in asthma because alterations in affinity of acetyltransferase for acetyl coA and in the regulation of enzyme activity may occur in acute asthma. Neutrophils from patients with acute asthma have increased levels of acetyltransferase activity which regulate production of platelet activating factor in inflammatory cells. Downregualtion of pyruvate dehydrogenase in VIP KO ice would therefore be a counter-regulatory mechanism [14].

### Extracellular superoxide dismutase

Superoxide dismutase decrease would explain the lack of protection from inflammation in VIP KO mice. It is consistent with the inability of the VIP KO mice to mount an anti-oxidant response. In agreement with Sackesen *et al*., [10] superoxide dismutase is decreased in asthmatic states in humans; we see this in our asthmatic VIP KO mice.

### Glycerol-3-phosphate

As a hypoxia-induced protein up-regulated under conditions of allergic airway inflammation, glycerol-3-phosphate dehydrogenase is a marker protein for allergic airway inflammation and its down-regulation in VIP KO mice may be part of a compensation response [15].

### The gene for carbonic anhydrase was upregulated in VIP KO mice

#### Carbonic anhydrase

Acetazolamide, a carbonic anhydrase inhibitor, increased the provocative concentration of a substance (methacholine) causing a 20% fall in the maximal amount of air forcefully exhaled in one second also known as the Forced Expired Volume in 1 second or FEV_1 _(log PC20) and was protective against sodium metabisulfite-induced challenge in mild asthma. Since carbonic anhydrase activity in the airways modulates bronchial hyperresponsiveness to metabisulfite challenge, the increased expression of carbonic anhydrase in VIP KO mice would be protective [15].

## Conclusions

This study presented a combined genomic and proteomic comparison of expression levels in WT and VIP KO mice. While revealing some striking changes, the data also reveal a generally poor correlation between chnages in transcriptomic and proteomic expression levels. This observation is consistent with other more extensive integrated studies of genomic and proteomic expression data that demonstrated that such data sets can be weakly correlated [16,17]. The reasons for this poor correlation may lie both in the nature of the analysis as well as the distinct regulatory mechanisms affecting transcriptomic and proteomic expression levels. The observation nonetheless underscores the importance of the combined approach.

These data support the concept that VIP mediates the pathogenesis of asthma by influencing the endogenous oxidant/antioxidant balance. VIP is resident in neural cells and mast cells and lymphocytes in humans. As an endogenous peptide, VIP's role in the oxidant/antioxidant balance is highlighted by up-regulation of carbonic anhydrase and a novel lung carbonyl reductase. Therapy with VIP therefore has justification, especially in those allergic asthmatics and in those whose disease is oxidant-rich such as severe asthmatics in exacerbation. Carbonic anhydrase was upregulated significantly according to gene microarray analysis and correlated with the clinical phenotype of airway hyperresponsiveness to methacholine in VIP KO mice; this is relevant as asthma in humans with mild reactions to sodium metabisulfite or sulfite sensitivity have increased levels of carbonic anhydrase. This lends support for VIP as a novel medicine to treat a subset of asthmatics who have airway hyperresponsiveness in particular to sodium metabisulfite which is ubiquitous in red wine, a product of the yeast *Saccaryomyces cerevisiae *in beer, and is often used in to preserve sundry foods.

## Authors' contributions

AS conceived of the study, designed and coordinated it and prepared lung specimens from VIP KO mice. DM carried out the proteomics analyses. TK analyzed proteomics data. SA evaluated genomics data. All authors read and approved the final manuscript.

## Supplementary Material

Additional File 1 **Table S1 - Upper panel gives the pixel density of the SSP spots in each of the eight gels (4 WT & 4KO)**. The lower panel gives the average data used in Table 1. Click here for file

## References

[B1] Szema AM Hamidi SA Lyubsky S Dickman KG Mathew S Abdel-Razek T Chen JJ Waschek JA Said SI Mice lacking the VIP gene show airway hyperresponsiveness and airway inflammation, partially reversible by VIP Am J Physiol Lung Cell Mol Physiol 2006 291 5 L880 6 Epub 2006 Jun 16 10.1152/ajplung.00499.2005 16782752

[B2] Colwell CS Disrupted circadian rhythms in VIP- and PHI-deficient mice Am J Physiol Regul Integr Comp Physiol 2003 285 R939 949 12855416 10.1152/ajpregu.00200.2003

[B3] Lotvall J Asthma endotypes: a new approach to classification of disease entities within the asthma syndrome J Allergy Clin Immunol 2011 127 355 360 10.1016/j.jaci.2010.11.037 21281866

[B4] Hamidi SA Prabhakar S Said SI Enhancement of pulmonary vascular remodelling and inflammatory genes with VIP gene deletion Eur Respir J 2008 31 135 139 10.1183/09031936.00105807 18166594

[B5] Ao X Comparative proteomic analysis of radiation-induced changes in mouse lung: fibrosis-sensitive and -resistant strains Radiat Res 2008 169 417 425 10.1667/RR1173.1 18363430

[B6] Peterson GL A simplification of the protein assay method of Lowry et al. which is more generally applicable Anal Biochem 1977 83 346 356 10.1016/0003-2697(77)90043-4 603028

[B7] Shevchenko A Wilm M Vorm O Mann M Mass spectrometric sequencing of proteins silver-stained polyacrylamide gels Anal Chem 1996 68 850 858 10.1021/ac950914h 8779443

[B8] Oppermann U Carbonyl reductases: the complex relationships of mammalian carbonyl- and quinone-reducing enzymes and their role in physiology Annu Rev Pharmacol Toxicol 2007 47 293 322 10.1146/annurev.pharmtox.47.120505.105316 17009925

[B9] Suzuki S Increase in reactive oxygen metabolite level in acute exacerbations of asthma Int Arch Allergy Immunol 2008 146 Suppl 1 67 72 18504410 10.1159/000126064

[B10] Sackesen C A comprehensive evaluation of the enzymatic and nonenzymatic antioxidant systems in childhood asthma J Allergy Clin Immunol 2008 122 78 85 10.1016/j.jaci.2008.03.035 18485467

[B11] Kamada F The GSTP1 gene is a susceptibility gene for childhood asthma and the GSTM1 gene is a modifier of the GSTP1 gene Int Arch Allergy Immunol 2007 144 275 286 10.1159/000106316 17643058

[B12] Fajardo I Svensson L Bucht A Pejler G Increased levels of hypoxia-sensitive proteins in allergic airway inflammation Am J Respir Crit Care Med 2004 170 477 484 10.1164/rccm.200402-178OC 15151919

[B13] Gilchrist M Hesslinger C Befus AD Tetrahydrobiopterin, a critical factor in the production and role of nitric oxide in mast cells J Biol Chem 2003 278 50607 50614 10.1074/jbc.M307777200 14514683

[B14] Misso NL Gillon RL Stewart GA Thompson PJ Lyso-PAF acetyltransferase activity in neutrophils of patients during acute asthma and after recovery Eur Respir J 1996 9 2243 2249 10.1183/09031936.96.09112243 8947067

[B15] O'Connor BJ Yeo CT Chen-Worsdell YM Barnes PJ Chung KF Effect of acetazolamide and amiloride against sodium metabisulphite-induced bronchoconstriction in mild asthma Thorax 1994 49 1096 1098 10.1136/thx.49.11.1096 7831623 PMC475268

[B16] Chen G Gharib TG Huang CC Taylor JM Misek DE Kardia SL Giordano TJ Iannettoni MD Orringer MB Hanash SM Beer DG Discordant protein and mRNA expression in lung adenocarcinomas Mol Cell Proteomics 2002 1 304 313 10.1074/mcp.M200008-MCP200 12096112

[B17] Tian Q Stepaniants SB Mao M Weng L Feetham MC Doyle MJ Yi EC Dai H Thorsson V Eng J Goodlett D Berger JP Gunter B Linseley PS Stoughton RB Aebersold R Collins SJ Hanlon WA Hood LE Integrated genomic and proteomic analyses of gene expression in Mammalian cells Mol Cell Proteomics 2004 3 960 969 10.1074/mcp.M400055-MCP200 15238602

