# Supplementary material for: Vasoactive Intestinal Peptide Knockout (VIP KO) mouse model of sulfite-sensitive asthma: up-regulation of novel lung carbonyl reductase
Source: BMC Immunol. 2011 Nov 21;12:66. doi: 10.1186/1471-2172-12-66 (PMC3277465; doi:10.1186/1471-2172-12-66)
Supplement: Additional File 1 — Table S1 - Upper panel gives the pixel density of the SSP spots in each of the eight gels (4 WT & 4KO). The lower panel gives the average data used in Table 1. [file 1471-2172-12-66-S1.DOC]

Additional File 1

Table S1

SSP Pixel Density Data

| SSP | ko 02250 | ko 02251 | ko 02252 | ko 02253 | wt 02254 | wt 02255 | wt 02256 | wt 02257 |
| --- | --- | --- | --- | --- | --- | --- | --- | --- |
| 3204 | 0 | 0 | 0 | 42275.4 | 112851 | 68487.8 | 76736.1 | 103066.4 |
| 3304 | 96027.2 | 63865 | 89490.8 | 39014.5 | 421999.2 | 197163 | 301249.1 | 329355 |
| 6304 | 0 | 0 | 78184.5 | 0 | 255580.2 | 107527.8 | 153548.6 | 76073 |
| 7105 | 24163.7 | 54333.8 | 26239 | 52468 | 0 | 0 | 0 | 0 |
| 7205 | 162309.2 | 388241.1 | 216941.7 | 123166.8 | 0 | 0 | 0 | 0 |
| 8101 | 175218.5 | 393313.5 | 158749.3 | 115803.7 | 0 | 0 | 11419 | 0 |
| 9206 | 306743.2 | 627033.4 | 990462.3 | 261099.9 | 523966.6 | 284216.5 | 757037.7 | 522101.1 |

| SSP | AV KO | AV WT | KO/WT | WT/KO | t-TEST |
| --- | --- | --- | --- | --- | --- |
| 3204 | 10568.85 | 90285.33 | 0.12 | 8.54 | 0.0018 |
| 3304 | 72099.38 | 312441.6 | 0.23 | 4.33 | 0.0108 |
| 6304 | 19546.13 | 148182.4 | 0.13 | 7.58 | 0.0377 |
| 7105 | 39301.13 | 0 | ∞ | 0.00 | 0.0171 |
| 7205 | 222664.7 | 0 | ∞ | 0.00 | 0.0318 |
| 8101 | 210771.3 | 2854.75 | 73.83 | 0.01 | 0.0440 |
| 9206 | 546334.7 | 521830.5 | 1.05 | 0.96 | 0.9049 |

Upper panel gives the pixel density of the SSP spots in each of the eight gels (4 WT & 4KO). The lower panel gives the average data used in TABLE 1.
